# Simplified dietary acute tryptophan depletion: effects of a novel amino acid mixture on the neurochemistry of C57BL/6J mice

**DOI:** 10.3402/fnr.v59.27424

**Published:** 2015-08-13

**Authors:** Cristina L. Sánchez, Amanda E. D. Van Swearingen, Andrew E. Arrant, Caroline S. Biskup, Cynthia M. Kuhn, Florian D. Zepf

**Affiliations:** 1 Clinic for Child and Adolescent Psychiatry, Psychosomatics and Psychotherapy, JARA Brain, RWTH Aachen University, Aachen, Germany; 2 Department of Pharmacology and Cancer Biology, Duke University Medical Center, Durham, NC, USA; 3 Department of Child and Adolescent Psychiatry, School of Paediatrics and Child Health & School of Psychiatry and Clinical Neurosciences, Faculty of Medicine, Dentistry and Health Sciences, The University of Western Australia, Perth, WA, Australia; 4 Specialised Child and Adolescent Mental Health Services (CAHMS), Department of Health, Perth, WA, Australia

**Keywords:** acute tryptophan depletion, dopamine, amino acid, formulation, mice, serotonin

## Abstract

**Background:**

Diet and nutrition can impact on the biological processes underpinning neuropsychiatric disorders. Amino acid (AA) mixtures lacking a specific neurotransmitter precursor can change the levels of brain serotonin (5-HT) or dopamine (DA) in the central nervous system. The availability of these substances within the brain is determined by the blood–brain barrier (BBB) that restricts the access of peripheral AA into the brain. AA mixtures lacking tryptophan (TRP) compete with endogenous TRP for uptake into the brain across the BBB, which in turn leads to a decrease in central nervous 5-HT synthesis.

**Objective:**

The present study compared the effects of a simplified acute tryptophan depletion (SATD) mixture in mice on blood and brain serotonergic and dopaminergic metabolites to those of a commonly used acute tryptophan depletion mixture (ATD Moja-De) and its TRP-balanced control (BAL).

**Design:**

The SATD formula is composed of only three large neutral AAs: phenylalanine (PHE), leucine (LEU), and isoleucine (ILE). BAL, ATD Moja-De, or SATD formulas were delivered to adult male C57BL/6J mice by gavage. TRP, monoamines, and their metabolites were quantified in blood and brain regions (hippocampus, frontal cortex, amygdala, caudate putamen, and nucleus accumbens).

**Results:**

Both ATD Moja-De and SATD significantly decreased levels of serum and brain TRP, as well as brain 5-HIAA and 5-HT compared with BAL. SATD reduced HVA levels in caudate but did not alter total DA levels or DOPAC. SATD decreased TRP and serotonergic metabolites comparably to ATD Moja-De administration.

**Conclusion:**

A simplified and more palatable combination of AAs can manipulate serotonergic function and might be useful to reveal underlying monoamine-related mechanisms contributing to different neuropsychiatric disorders.

Dietary amino acid (AA) depletion mixtures have been used in neuropsychiatric research (1) to study the underlying role of monoamine neurotransmitters (such as serotonin [5-HT], dopamine [DA], or norepinephrine [NE]) in behavioral and psychiatric disorders (i.e. depression), and also (2) as potential nutritional challenge strategies for these disorders (1, 2). The underlying methodological factors that determine the neurochemical response to these particular AA mixtures have been discussed, and two major areas of current interest are the development of simplified mixtures that achieve the same results with greater tolerability as well as the investigation of specific neural mechanisms mediating behavioral changes associated with these treatments. The specific mixture composition is one variable which can influence the consequences of these challenge procedures on neurotransmitter synthesis in the brain (3–7).

Biogenic amines (indoleamines, catecholamines, and histamine) are produced from only three essential AAs: tryptophan (TRP), tyrosine (TYR), and histidine (HIS). From embryonal and fetal development, these neurotransmitters control and regulate various vital functions. Changes in monoamine neurotransmission can modify neural function at multiple levels, such as growth and development at early stages and sexual development during maturity (8, 9). Thus, when serum TRP is increased by a higher intake through diet, an immediate boost effect is produced on synthesis of 5-HT in the enterochromaffin cells of the GI tract and in the brain. On the contrary, when TRP intake is lower with diet, a depletion effect on peripheral and central 5-HT will be produced (7). These effects are measurable even during fetal and neonatal development (8).

Lowering the concentration of specific AAs that serve as precursors for neurotransmitters in the brain to transiently lower synthesis and release of the neurotransmitter under investigation can also be used to elucidate behavioral and neurobiological effects of the neurotransmitter in question (10–12). This method has been particularly effective when studying 5-HT, as tryptophan hydroxylase 2 (TPH-2, the rate-limiting enzyme for central nervous synthesis of 5-HT) is not saturated at circulating levels of TRP and therefore manipulating TRP availability significantly influences 5-HT synthesis and release (13). Although less effective, a similar strategy has shown some success in transiently lowering availability of TYR and PHE for catecholamine synthesis (2, 14–17).

The basic mechanism underpinning this strategy is that large neutral AAs (LNAAs) compete with each other for transport across the blood–brain barrier (BBB) using a shared transporter (LAT-1), and so changing the ratio of competing AAs (CAAs) can limit the entry of precursor AAs into the brain (18, 19). Thus, access of each CAA to the brain depends not only on its serum levels but also on the serum concentration of the other LNAAs competing for transport. Phenylalanine (PHE) and leucine (LEU) have a lower K_m_ for transport across the BBB than TRP (20). These particular AAs should provide optimal competition with TRP for transport into the brain. Alteration of peripheral protein synthesis after AA administration may also contribute to lowering brain TRP availability, since administration of an AA mixture stimulates protein synthesis in the liver, depleting plasma TRP, and thus favoring entry of CAAs with consequent reduction of brain TRP (21). Moreover, branch-chained amino acids (BCAAs) and LEU, in particular, may stimulate skeletal muscle protein synthesis (22).

Much effort had been made to identify the optimal composition of these dietary AA-based depletion formulations. Young et al. (23) adopted a composition based on the human milk profile, containing up to 15 AAs (both essential and non-essential AAs). This formulation was widely accepted and used in many studies in the past. However, this very complex mixture presents some disadvantages, including nauseating taste due to the sulfur-containing AAs and low specificity of the targets due to a high concentration of BCAAs (24). The proportions of the complex mixtures were subsequently modified by other groups (25–30) to optimize depletion. The core features of these dietary mixtures formed by the neurotransmitter precursors (TRP, PHE, and TYR) and BCAAs (valine [VAL], leucine [LEU], and isoleucine [ILE]) have been never altered. However, Badawy et al. (24, 31) postulated that decreasing the content of BCAAs might improve target specificity. Many of these investigators have modified the AA profile of their formulations or studied the use of proteins such as gelatin or α-lactalbumin (32–35).

We have previously shown that a modified version of a dietary ATD protocol termed Moja-De that minimizes content of sulfur-containing AAs causes less nausea and discomfort in people but decreases 5-HT synthesis in a mouse model as much as the originally suggested formulation (36). While this new dietary mixture offers an advantage in human studies, it is still quite complex in its formulation (Table 1) and potentially includes off-target responses due to large neutral AAs. In the present study, we tested the hypothesis that providing a simplified dietary AA formula (SATD, Simplified Acute Tryptophan Depletion) which includes just three AAs to compete at LAT-1 with TRP, will subsequently lower central nervous 5-HT synthesis. We investigated variations in serum and various brain regions of TRP and 5-HT metabolite contents, as well as TYR and DA and their metabolite levels and compared them with a control condition formula (BAL) as well as a positive control (ATD Moja-De).

**Table 1:** Quantities of amino acids for each mixture

	BAL		ATD		SATD	
			
Amino acids	mg/kg	mg/25 g (mouse)	mg/kg	mg/25 g (mouse)	mg/kg	mg/25 g (mouse)
Phenylalanine	132	3.30	132	3.30	300	7.5
Leucine	132	3.30	132	3.30	1280	32
Isoleucine	84	2.10	84	2.10	250	6.25
Methionine	50	1.25	50	1.25	–	–
Valine	96	2.40	96	2.40	–	–
Threonine	60	1.50	60	1.50	–	–
Lysine-HCl	120	3.00	120	3.00	–	–
Tryptophan	70	1.75	–	–	–	–
Total content	744	18.6	674	15.3	1,830	45.8

BAL=balanced, control condition; ATD=acute tryptophan depletion; SATD=simplified acute tryptophan depletion.

## Materials and methods

### Animals

Adult male mice (8–9 weeks old) from Jackson Laboratories (Bar Harbor, ME, USA) were housed in groups of eight per cage in an approved animal facility under controlled temperature (21±1°C) and humidity with a 12-h light–dark cycle (lights on at 6 a.m.). The animals were allowed to acclimate for 1 week upon arrival; food (LabDiet^®^) and water were freely provided until the night prior to the experiment, when food was removed. Mice were handled daily to habituate to handling. On the day of testing, food-deprived mice received an AA mixture (BAL, ATD, or SATD) by gavage. The animals were anesthetized with isoflurane and killed by decapitation 2.5 h after the administration of the corresponding formula. The Animal Care and Use Committee at Duke University Medical Center approved this experimental protocol.

### The new AA formula

The present study introduces a simplified dietary acute tryptophan depletion formula (SATD), which is based on the previously reported ATD Moja-De mixture (29, 36), but is composed of only three LNAAs: PHE, LEU, and ILE. These AAs compete with TRP for access into the brain and thereby provide a TRP-free mixture of LNAAs that lowers central nervous system 5-HT synthesis. To provide adequate concentrations of CAAs, the total amount of each AA was increased relative to the original formula. SATD administration was compared with a control mixture containing a complex mixture of amino acids (BAL), and to the Moja-De ATD modification of the TRP-free mixture. Table 1 shows the quantities of the components of the different dietary mixtures. The AA mixtures were prepared by the Pharmacy of the University Hospital of RWTH Aachen University (Germany). They were mixed in deionized water on the morning of the experiment, and polytron and sonication bath were used until the formula was completely suspended. The AA mixtures were administered in two doses spaced 30 min apart by gavage with 2 g/kg BW mixed with 6.66 mL/kg BW deionized water, for a total dose of 4 g/kg.

### Blood and tissue preparation

Blood samples were collected by cardiac puncture and spun at 16,000×g and 4°C for 20 min. Aliquots of the supernatant were frozen at −80°C until the day of analysis by high-performance liquid chromatography (HPLC). Samples were thawed on the day of assay and 10 µL of serum was diluted with 990 µL of ice-cold standard buffer (0.5 mM sodium metabisulfite, 0.2 N perchloric acid and 0.5 mM EDTA) and spun at 16,000×g and 4°C for 10 min.

Different brain regions (hippocampus, frontal cortex, amygdala, caudate putamen, and nucleus accumbens) were dissected on ice using a mouse brain block, weighed, placed in 1.5 mL Eppendorf tubes and immediately frozen at −80°C. To quantitate the AAs TRP and TYR, as well as the monoamines and their metabolites, brain samples were homogenized in 250 µL of ice-cold standard buffer, except for the caudate samples that homogenized in 500 µL of standard buffer due to the high DA content. The whole homogenates were disrupted by sonication and centrifuged at 16,000×g and 4°C for 20 min. The supernatants were collected and kept on ice, until analysis.

### Quantifying the analytes

To analyze the AAs TRP and TYR, a BAS LC-4B apparatus with an electrochemical detector with dual 3 mm carbon electrode (MF-1000) and reference electrode (MF-2021) was used. TRP separation was conducted on a C18 column (Phenomenex^®^ Kinetex™ 2.6 µm, 100×4.6 mm, 100 Å) using the following mobile phase: 8% acetonitrile (v/v), 0.05 M citric acid, 0.05 M, Na_2_HPO_4_·7H_2_O and 0.1 mM EDTA and a flow rate of 1.0 mL/min. Mobile phase was not corrected for pH. Samples were quantified by electrochemical detection with the detector set to 0.85 V versus Ag/AgCl reference electrode and sensitivity at 200 nA. TYR separation was performed on the same type of column with a mobile phase comprising the following: 12% methanol (v/v), 0.1 M, NaH_2_PO_4_·7H_2_O, 0.1 mM EDTA and 2.7 mM octanesulfonic acid (anhydrous); pH 3.8 and a flow of 1.2 mL/min. For electrochemical detection, the detector was set to 0.80 V, sensitivity at 950 nA.

Dopaminergic (DA, DOPAC, and HVA) and serotonergic (5-HT and 5-HIAA) contents were determined using a Constametric^®^ 4100 apparatus, C18 column (Phenomenex^®^ Kinetex™ 2.6 µm, 100×4.6 mm, 100 Å and an electrochemical detector with dual 3 mm carbon electrode (MF-1000) and reference electrode (MF-2021). The mobile phase used for separating these metabolites consisted of: 18% methanol (v/v), 0.1 M sodium phosphate, 0.8 mM octanesulfonic acid (anhydrous), and 0.1 mM EDTA; pH 3.1. Flow rate at 0.7 mL/min. Detector was set to 0.70 V and sensitivity at 50 nA.

### Statistical analysis

Statistical evaluation of the data was carried out using NCSS 2007 software (NCSS LLC, Kaysville, UT, USA). Effects of treatment for every dependent measure (TRP, TYR, 5-HT, 5-HIAA, DA, DOPAC and HVA) were analyzed for all groups (BAL, ATD, SATD) by a repeated measures global ANOVA with ‘treatment’ as a between factor and ‘serum’ or ‘brain region’ as a within factor. Analyses that yielded significant main effects and/or interactions were subjected to a lower-order two-way ANOVA and significant two-way interactions were followed by Fisher's LSD multiple-comparison *post-hoc* testing with the level of significance set at *p<*0.05. All data are shown as mean±S.E.M. (Table 2). Outliers were determined by a GRUBBS statistical test.

**Table 2:** Contents of TRP, TYR, serotonergic (5-HT and 5-HIAA), and dopaminergic (DA, DOPAC and HVA) metabolites in the serum and different brain regions of mice

	Serum	Hippocampus	Frontal cortex	Amygdala	Caudate putamen	Nucleus accumbens
Tryptophan						
BAL	26.0±2.1	3.6±0.4	3.1±0.1	4.0±0.1	4.3±0.1	3.4±0.1
ATD	16.0±1.9*	2.0±0.4*	1.0±0.1*	1.9±0.1*	2.0±0.1*	1.7±0.1*
SATD	18.0±1.9*	2.1±0.4*	1.5±0.1*	2.0±0.1*	2.3±0.1*	1.8±0.1*
5-HT						
BAL	1.6±0.4	0.43±0.02	0.24±0.01	0.52±0.02	0.36±0.01	0.46±0.02
ATD	1.8±0.3	0.36±0.02*	0.19±0.01*	0.36±0.02*	0.29±0.01*	0.33±0.02*
SATD	1.7±0.3	0.35±0.02*	0.21±0.01	0.39±0.02*	0.30±0.01*	0.36±0.02*
5-HIAA						
BAL	n.a.	0.66±0.03	0.18±0.01	0.42±0.02	0.48±0.02	0.44±0.02
ATD	n.a.	0.29±0.03*	0.12±0.01*	0.23±0.02*	0.23±0.02*	0.22±0.02*
SATD	n.a.	0.37±0.03*	0.11±0.01*	0.24±0.02*	0.25±0.02*	0.26±0.02*
5-HIAA/5-HT						
BAL	n.a.	1.5±0.01	0.77±0.07	0.81±0.07	1.29±0.04	0.97±0.06
ATD	n.a.	0.8±0.01*	0.66±0.06	0.68±0.07	0.79±0.04*	0.70±0.05*
SATD	n.a.	1.1±0.01*	0.53±0.07	0.64±0.08	0.83±0.05*	0.73±0.06*
Tyrosine						
BAL	7.5±0.9	4.0±0.4	3.5±0.2	3.7±0.3	2.8±0.3	4.1±0.4
ATD	5.5±0.9	3.3±0.4	2.7±0.3	2.8±0.3	1.9±0.2	3.0±0.4
SATD	7.6±0.9	3.3±0.4	2.7±0.3	2.7±0.3	2.1±0.3	3.1±0.4
DA						
BAL	n.a.	0.01±0.01	0.05±0.01	0.25±0.02	10.1±0.6	4.3±0.4
ATD	n.a.	0.01±0.01	0.05±0.01	0.24±0.02	10.0±0.5	3.5±0.4
SATD	n.a.	0.01±0.01	0.04±0.01	0.30±0.02	10.2±0.6	3.5±0.4
DOPAC						
BAL	n.a.	0.01±0.01	0.04±0.01	0.06±0.01	2.1±0.2	1.2±0.1
ATD	n.a.	0.01±0.01	0.04±0.01	0.07±0.01	1.9±0.2	1.1±0.1
SATD	n.a.	0.01±0.01	0.03±0.01	0.09±0.01*	1.9±0.2	1.1±0.1
HVA						
BAL	n.a.	0.04±0.002	0.14±0.01	0.16±0.01	2.2±0.01	1.2±0.07
ATD	n.a.	0.03±0.002*	0.12±0.01	0.15±0.01	1.9±0.01*	1.0±0.06
SATD	n.a.	0.03±0.002*	0.11±0.01	0.16±0.01	1.7±0.01*	0.9±0.07*
HVA/DA						
BAL	n.a.	3.8±3.5	3.0±0.7	0.64±0.06	0.22±0.01	0.29±0.02
ATD	n.a.	5.1±3.2	3.9±0.7	0.68±0.06	0.19±0.01	0.31±0.02
SATD	n.a.	10.8±3.2	3.3±0.8	0.52±0.06	0.18±0.01	0.26±0.02

Values are means±S.E.M. of 6–8 animals/treatment and expressed in µg/mL (serum samples) and ng/mg (tissue samples).

* indicates *p*<0.05 versus balanced; n.a. indicates not applicable.

## Results

### SATD affects TRP content in serum

**Fig. 1:**
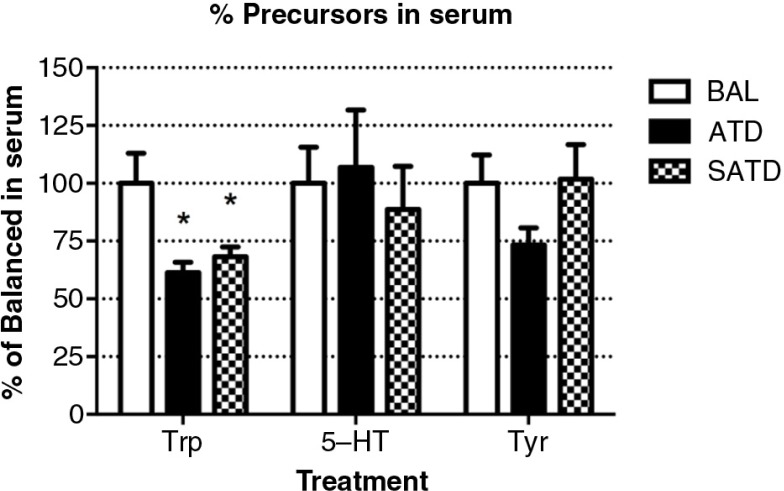
Serum levels of tryptophan (TRP), serotonin (5-HT), and tyrosine (TYR) in the mouse after formula administration. Data are represented as mean±S.E.M. Groups of 7–8 mice received either a control condition (BAL), acute tryptophan depletion (ATD), or simplified acute tryptophan depletion (SATD) mixtures. *p<0.05 compared with BAL.

The contents of the AAs TRP and TYR and the neurotransmitter 5-HT were measured in serum of male C57BL/6J mice treated with BAL, ATD, or SATD (Fig. 1). Animals treated with either ATD or SATD showed significantly decreased serum TRP content relative to controls (*F*_[2,23]_=6.85, *p=*0.005). However, no treatment effect of ATD or SATD on serum TYR was detected (*F*_[2,23]_=1.86, *p=*0.181). Circulating 5-HT concentrations were not significantly different between either SATD or ATD treatments versus BAL administration (*F*_[2,23]_=0.04, *p=*0.965).

### Robust reductions in TRP and serotonergic metabolite levels in brain tissue

**Fig. 2:**
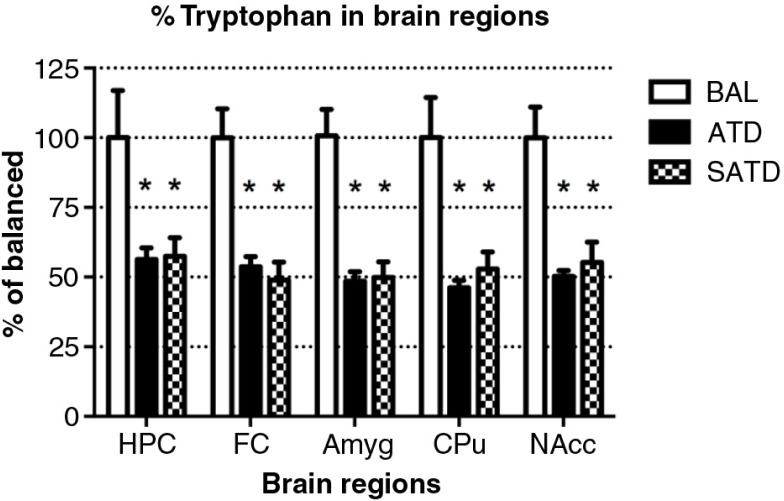
Levels of tryptophan in the different brain regions of the mouse after formula administration. Data are represented as mean±S.E.M. Groups of 7–8 mice received either a control condition (BAL), acute tryptophan depletion (ATD), or simplified acute tryptophan depletion (SATD) mixtures. HPC: hippocampus; FC: frontal cortex; Amyg: amygdala; CPu: caudate putamen; NAcc: nucleus accumbens. *p<0.05 compared with BAL.

**Fig. 3:**
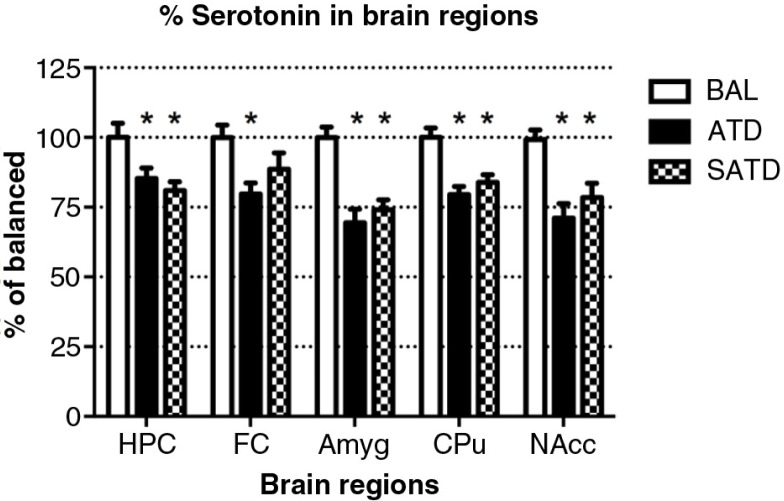
Levels of serotonin (5-HT) in the different brain regions of the mouse after formula administration. Data are represented as mean±S.E.M. Groups of 7–8 mice received either a control condition (BAL), acute tryptophan depletion (ATD), or simplified acute tryptophan depletion (SATD) mixtures. HPC: hippocampus; FC: frontal cortex; Amyg: amygdala; CPu: caudate putamen; NAcc: nucleus accumbens. *p<0.05 compared with BAL.

**Fig. 4:**
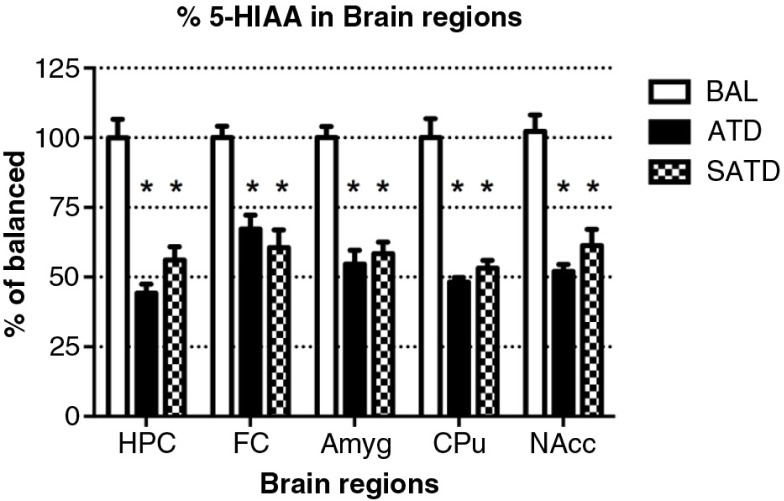
Levels of 5-hydroxyindoleacetic acid (5-HIAA) in the different brain regions of the mouse after formula administration. Data are represented as mean±S.E.M. Groups of 7–8 mice received either a control condition (BAL), acute tryptophan depletion (ATD), or simplified acute tryptophan depletion (SATD) mixtures. HPC: hippocampus; FC: frontal cortex; Amyg: amygdala; CPu: caudate putamen; NAcc: nucleus accumbens. *p<0.05 compared with BAL.

A significant decrease of brain TRP levels was shown after SATD and ATD administration (*F*_[2,109]_=13.98, *p<*0.001; *F*_[4,109]_=12.97, *p<*0.0001, for an effect of treatment and region, respectively). A global ANOVA showed a main effect of treatment, region, and treatment×region on brain 5-HT and its metabolite 5-HIAA (Treatment: *F*_[2,108]_=41.11, *p<*0.0001; *F*_[2,108]_=50.44, *p<*0.0001; region: *F*_[4,108]_=65.79, *p<*0.0001; *F*_[4,108]_=211.50, *p<*0.0001; treatment×region: *F*_[8,108]_=2.40, *p=*0.023; *F*_[8,108]_=20.23, *p<*0.0001, respectively). Fisher's *post-hoc* tests showed the reductions in the brain 5-HT and 5-HIAA contents of mice after dietary SATD or ATD treatment relative to control condition (see Figs. 2–4 for a summary). While brain 5-HT concentrations were reduced by 20–35% after dietary administration of both SATD and ATD (Table 3) when compared with BAL, 5-HIAA exhibited a significantly higher decline (around 40–55%, *p<*0.0001). Globally, dietary administration of the new SATD mixture caused greater 5-HT-related depletion magnitude when compared with the ATD Moja-De formula (Table 2).

**Table 3:** This is a summary of the effects of the treatments relative to balanced mixture (BAL)

	Serum analytes			Brain serotonergic system			Brain dopaminergic system			
			
Treatment	TRP	5-HT	TYR	Trp	5-HT	5-HIAA	TYR	DA	DOPAC	HVA
ATD	↓↓	=	=	↓↓	↓	↓↓	=	=	=	↓ (HPC, CPu)
SATD	↓↓	=	=	↓↓	↓	↓↓	=	=	↓ (Amyg)	↓ (HPC, NAcc, CPu)

FC=frontal cortex; HPC=hippocampus; Amyg=amygdala; CPu=caudate putamen. For effects that were observed only in certain regions, the affected region is indicated in parenthesis. ↓↓ indicates a 35–60% depletion; ↓ indicates a 20–34% depletion;**=**indicates no effects.

### Brain HVA decreased after administration of ATD or SATD

**Fig. 5:**
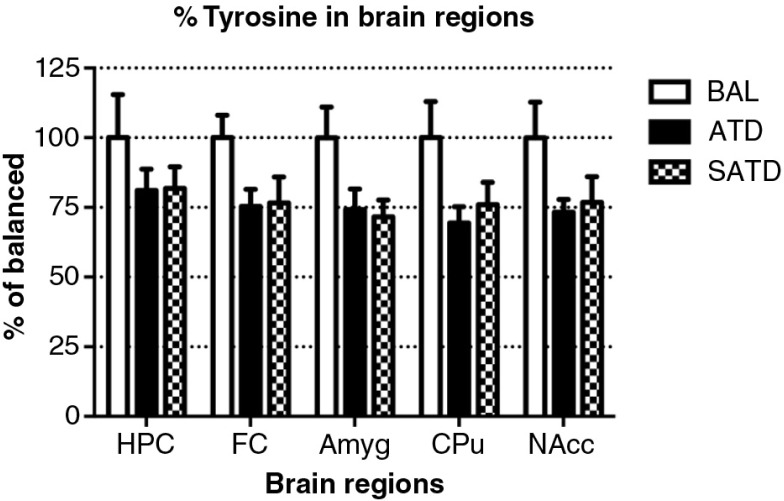
Levels of tyrosine in the different brain regions of the mouse after formula administration. Data are represented as mean±S.E.M. Groups of 7–8 mice received either a control condition (BAL), acute tryptophan depletion (ATD), or simplified acute tryptophan depletion (SATD) mixtures. HPC: hippocampus; FC: frontal cortex; Amyg: amygdala; CPu: caudate putamen; NAcc: nucleus accumbens.

**Fig. 6:**
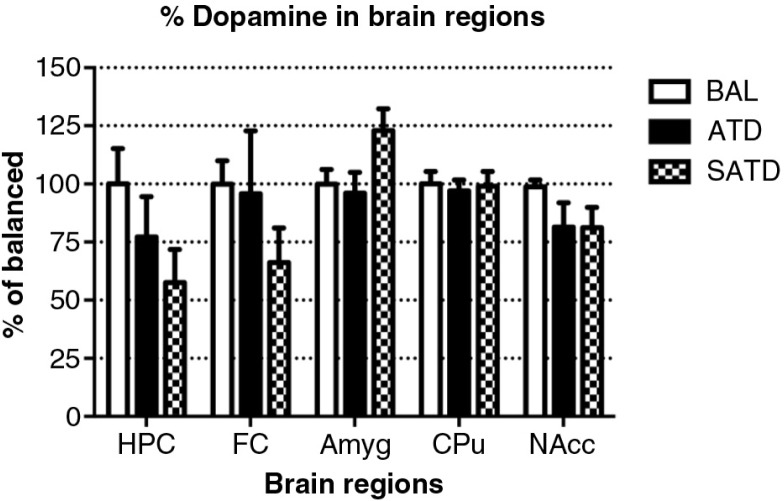
Levels of dopamine (DA) in the different brain regions of the mouse after formula administration. Data are represented as mean±S.E.M. Groups of 7–8 mice received either a control condition (BAL), acute tryptophan depletion (ATD), or simplified acute tryptophan depletion (SATD) mixtures. HPC: hippocampus; FC: frontal cortex; Amyg: amygdala; CPu: caudate putamen; NAcc: nucleus accumbens.

**Fig. 7:**
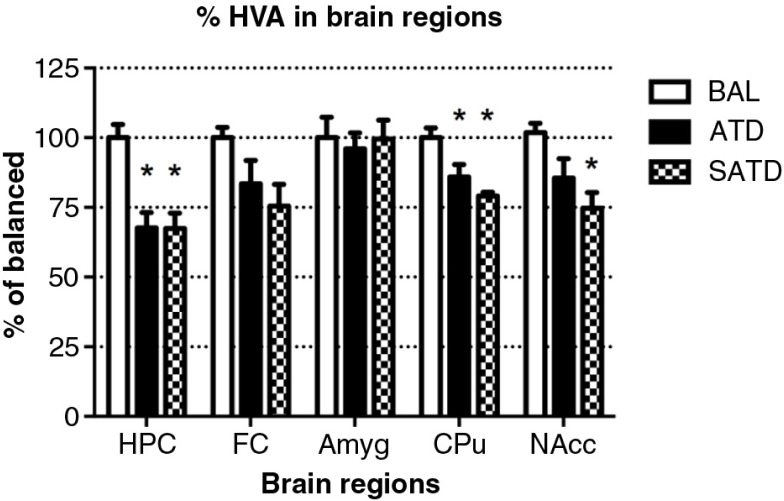
Levels of homovanillic acid (HVA) in the different brain regions of the mouse after formula administration. Data are represented as mean±S.E.M. Groups of 7–8 mice received either a control condition (BAL), acute tryptophan depletion (ATD), or simplified acute tryptophan depletion (SATD) mixtures. HPC: hippocampus; FC: frontal cortex; Amyg: amygdala; CPu: caudate putamen; NAcc: nucleus accumbens. *p<0.05 compared with BAL.

There was no main effect of treatment on brain TYR, DA, or DOPAC (*F*_[2,109]_=2.78, *p=*0.087; *F*_[2,106]_=2.06, *p=*0.154; *F*_[2,106]_=0.24, *p=*0.788, respectively, Figs. 5 and 6). However, a global ANOVA indicated an effect of region in both analytes (TYR: *F*_[4,109]_=31.64, *p<*0.0001; DA: *F*_[4,108]_=518.73, *p<*0.0001; DOPAC: *F*_[4,108]_=302.21, *p<*0.0001). A lower-order ANOVA and Fisher's *post-hoc* tests indicated that the dopaminergic metabolite DOPAC in the amygdala of SATD-treated mice was significantly higher relative to BAL-treated mice (*F*_[2,21]_=4.91, *p=*0.020). HVA showed a significant effect of treatment, region and treatment×region (Treatment: *F*_[2,107]_=6.29, *p=*0.008; region: *F*_[4,107]_=1349.35, *p<*0.0001; treatment×region: *F*_[8,107]_=7.23, *p<*0.001). The levels of HVA were affected (25–35% depletion, Table 3) in hippocampus, caudate, and nucleus accumbens when SATD was administered. ATD administration reduced HVA content in hippocampus and caudate (Fig. 7).

### Effect on 5-HT and DA turnovers

5-HT turnover (5-HIAA/5-HT ratio) presented a significant effect of treatment (*F*_[2,108]_=11.17, *p<*0.001), region (*F*_[4,108]_=62.51, *p<*0.0001), and a treatment×region interaction (*F*_[8,108]_=9.87, *p<*0.0001, Table 2). These values showed a reduced ratio after dietary SATD and ATD administration when compared with the BAL condition in the hippocampus, caudate, and nucleus accumbens. Moreover, DA turnover (HVA/DA ratio) did not exhibit a main effect of treatment but of region (*F*_[2,106]_=1.45, *p=*0.260; *F*_[4,106]_=10.78, *p<*0.001, respectively). An increase in this ratio was achieved with SATD and ATD treatments in the hippocampus and frontal cortex compared with other regions, as indicated by a lower-order ANOVA and Fisher's *post-hoc* tests.

## Discussion

Previous studies from our group have demonstrated that the ATD formula Moja-De or combined monoamine depletion mixture (CMD) successfully reduce TRP and serotonergic metabolite levels in blood and brain tissues (29, 36). The present study demonstrates the efficacy of a simplified dietary formulation in affecting serum and different brain AA and neurotransmitter metabolite contents in a mouse model. These findings suggest that SATD, which is composed of only three essential AAs (PHE, LEU, and ILE), can compete with TRP at the BBB sufficiently to significantly impair its transport. SATD induced a robust decrease in serum and brain TRP levels of male C57BL/6J mice compared with control condition (BAL) and these results are in line with those obtained with ATD. Moreover, significant reductions in brain 5-HT and its metabolite 5-HIAA caused after SATD administration were equivalent to those induced by ATD Moja-De relative to BAL (Table 2) and greater than those induced by CMD in our previous study (29). SATD reduced the final product of this pathway (HVA); however, no changes were observed either on the AA precursor TYR nor the subsequent molecules DA or DOPAC. One explanation for this finding could be that DA turnover was affected indirectly by altered serotonergic turnover. These results support the potential utility of this simplified combination of AAs to examine the neurochemical underpinnings of different neuropsychiatric disorders.

The AA content of the new dietary SATD mixture is dramatically simplified from the original Moja-De ATD (28). The AA composition was reduced from 7 to 3 essential AAs (compensated by an increased amount of each). PHE, LEU and ILE were selected for this formulation based on their reported affinity for the transporter LAT-1 and concentration in the plasma to provide optimal competition for TRP transport into the central nervous system (37, 38). It is unlikely that peripheral effects on protein synthesis are the critical determinants for successful TRP depletion in the brain and successful transient inhibition of serotonergic function. While it will be important to investigate the neurochemical outcome of this mixture further, the present findings suggest that this simpler formulation may present two advantages for effecting TRP depletion in studies focusing on serotonergic effects on behavior and disease, in particular because a simpler mixture is available that may be better tolerated by humans, and a mixture with fewer off-target effects due to the lack of LNAAs.

A drawback of this new mixture might be the higher total dose of AAs used in this particular experiment, which may lead to a reduced tolerability in humans. High doses of AAs have been used with conventional ATD mixtures, some applying up to 100 g of AAs in one dose (23). However, since these mixtures contained a different composition of AAs, and the observed clinical effects as well as side effects cannot be directly compared. To optimize the dosing regimen, a comparison of different doses should be conducted to find an optimal dose–response relationship before testing this mixture in humans and to allow for the smallest effective dose possible and thereby minimize possible side and adverse effects.

Another possible pitfall of this novel formulation may be its main attribute, namely the reduced number of AAs used. PHE, which is the essential AA precursor of tyrosine and comprises a relative larger ratio of SATD as compared with conventional ATD mixtures, might increase DA synthesis. It has been used as a challenge test in neurological research in a diagnostic setting (39, 40). Patients with dopa-responsive dystonia (DRD), who have a significantly different PHE/TYR ratio as compared with other dystonic patients, showed an improvement of symptoms after a PHE-challenge with 100 mg PHE/kg bodyweight (39, 40). Taking into account that the amount of PHE applied with the SATD is relatively larger than the conventional ATD mixtures, and the amount of AA to compete with PHE's uptake across the BBB is relatively small, one possible result could be an increase in dopaminergic synthesis in some brain areas. This aspect of SATD needs to be further investigated, ideally in brain areas typically densely populated with dopaminergic neurons.

Another consideration is that although this mixture is simplified, it contains BCAA, which contributes to off-target effects. BCAA may contribute to the change in DA and possibly glutamate after TRP depletion, and behavioral consequences of these changes (24, 31, 41, 42). In addition, BCAA have both central and peripheral effects on glucose and protein metabolism that can indirectly change brain function (43).

Dietary TRP depletion affects mood, behavior and cognition in many species (26, 28, 29, 36, 44). Its great value is that changes are most notable in specific vulnerable patient populations (12, 27, 45). Although TRP depletion does not lower mood in most healthy individuals, volunteer participants – in particular women – with a past history of depression or bipolar illness or family history of depression can exhibit decreased mood after dietary TRP depletion (12, 46–48). More recent research has shown that changes in emotional and cognitive processing occur when even small changes in central nervous system TRP levels occur in vulnerable populations (30, 49). However, many formulations are complex to prepare, and nauseating to subjects. The present results have identified a dietary formulation (SATD) with a very limited number of AAs (only three of them) that achieved a level of depletion previously obtained with a standard ATD protocol. Such combinations may provide a useful tool for future studies of neurochemical mechanisms related to the neurobiological underpinnings of neuropsychiatric disorders in terms of a neurodietary approach.

The use of AA-based depletion paradigms is also of clinical relevance. One possible target could be seen in the use of ATD protocols with regard to manic symptoms, which are at least in part thought to be related to a hyper-serotonergic state (50–52). For instance, one double-blind, placebo-controlled pilot study using ATD in acutely manic patients showed that patients who ATD in combination with sodium valproate showed greater improvement in mania ratings (53). However, a high intolerance rate limited the use of the used depletion paradigm. However, with regard to recent research using more refined and body weight–adapted depletion paradigms such as the Moja-De ATD protocol tolerability was at acceptable rates (54, 55), and neurodietary depletion strategies such as the ATD technique can provide important information on brain function and the neurochemical modulation of neurocircuitries in patients with psychiatric disorders and healthy people (53–57).

In conclusion, the present study has successfully demonstrated the action of a new simplified dietary AA formulation as regards impacting brain 5-HT content in the same way as the previously used ATD Moja-De depletion protocol (with a more complex AA-related content) does.
